# RecMotif: a novel fast algorithm for weak motif discovery

**DOI:** 10.1186/1471-2105-11-S11-S8

**Published:** 2010-12-14

**Authors:** He Quan Sun, Malcolm Yoke Hean Low, Wen Jing Hsu, Jagath C Rajapakse

**Affiliations:** 1 School of Computer Engineering, Nanyang Technological University, 639798, Singapore

## Abstract

**Background:**

Weak motif discovery in DNA sequences is an important but unresolved problem in computational biology. Previous algorithms that aimed to solve the problem usually require a large amount of memory or execution time. In this paper, we proposed a fast and memory efficient algorithm, RecMotif, which guarantees to discover all motifs with specific (*l*, *d*) settings (where *l* is the motif length and *d* is the maximum number of mutations between a motif instance and the true motif).

**Results:**

Comparisons with several recently proposed algorithms have shown that RecMotif is more scalable for handling longer and weaker motifs. For instance, it can solve the open challenge cases such as (40, 14) within 5 hours while the other algorithms compared failed due to either longer execution times or shortage of memory space. For real biological sequences, such as *E.coli CRP*, RecMotif is able to accurately discover the motif instances with (*l*, *d*) as (18, 6) in less than 1 second, which is faster than the other algorithms compared.

**Conclusions:**

RecMotif is a novel algorithm that requires only a space complexity of *O*(*m*^2^*n*) (where *m* is the number of sequences in the data and *n* is the length of the sequences).

## Introduction

After transcription and translation, genetic information in gene sequences that will be passed to offsprings is expressed into functional products such as proteins. Before transcription, transcriptional factors bind to DNA sequences to regulate the transcription of DNA into mRNA. The domains where the transcriptional factors are bound are called as *Transcriptional Factor Binding Sites* (*TFBSs*), also known as DNA motifs. To understand the complex regulatory process, the first task is to locate these *TFBSs*, i.e. DNA motif discovery [1].

An algorithm challenge of DNA motif discovery was proposed in [1]: find a motif of length *l* in *m* DNA sequences. Each sequence is *n* nucleotides long and contains one motif instance with up to *d* mutations to the true motif. In the above *Motif Challenge Problem* (*MCP*), typical values of *l*, *d*, *m*, *n* are 15, 4, 20 and 600. Following the definitions here, we model a motif as (*l*, *d*). For a specific *l*, the larger the value *d* and the longer the sequences, the weaker the motif thus the more difficult to discover it.

For the (15, 4) motif, probabilistic algorithms, such as MEME [2] and GibbsDNA [3], cannot give satisfactory performance with *n>*400 [1].

WINNOWER is a combinatorial approach which uses all sample substrings in the data set to discover cliques of motif instances, i.e. it is a sample-driven algorithm. It works by deleting spurious edges between vertices in a constructed graph, where the vertices represent the *l*-mer substrings in the data set and the edges indicate that the hamming distances between two vertices are not more than 2*d*. The final edges left after deletions are expected as the edges between the motif instances. That is, the remaining vertices are the motif instances. WINNOWER shows better results than MEME and GibbsDNA. However, substantial memory and execution time are required by WINNOWER [1].

PROJECTION is also a sample-driven algorithm using random projection and *Expectation Maximization* (*EM*) [4]. Random projection is the key step, which prepares starting points for *EM*. In this step, PROJECTION maps all the *l*-mer substrings from the data into buckets labeled with *k*-mer subsequences of the substrings. After projection, if a bucket holds a significant number of projected substrings, a *Position Weight Matrix* (*PWM*) is formed using the related substrings. With such *PWMs*, it is expected that *EM* has a higher chance to find the true motif. PROJECTION successfully tackled the extensions of *MCP* such as (16, 5) and (18, 6). However, the performance of PROJECTION decreases as the sequence length *n* is increased [4].

DPCFG is a sample-driven approach that finds cliques of the motif instances in graphs [9]. According to each of the *l*-mer substrings (reference substring) from one random reference sequence, it sifts out all the substrings within 2*d* distance in the remaining sequences and constructs lists for each of the substrings. A dynamic strategy is used in the construction: lists on substrings of sequence *i* will be constructed only using lists on substrings of sequence *i-*1. DPCFG has been shown to be efficient compared with several popular motif discovery algorithms. However, DPCFG has difficulty in handling highly degenerate motifs such as (24, 8). It requires substantial memory resources when dealing with weaker motifs because of the many intermediate lists of small sizes it needs to maintain.

PMSprune is a pattern-driven algorithm developed from *PMS* series (*Planted Motif Search*) [5-7]. It generates and checks the *d*-neighbors of each substring of length *l* sampled from a reference sequence. For each generated *l*-mer string, if it can find at least one substring from each of the other sequences such that their hamming distance is not more than *d*, the string can be output as a true motif. PMSprune puts the *d*-neighbors of a *l*-mer string in a tree structure. With a branch and bound strategy, it avoids checking all of the *d*-neighbors in the tree, which reduces the running time. For motifs with short *l*, PMSprune works well. However, it has difficulty in dealing with longer and weaker motifs.

iTriplet is also a pattern-driven algorithm [8]. In iTriplet, two random sequences are selected as the reference sequences. Then all combinations of two *l*-mer substrings respectively from the two reference sequences are used together with each of the substrings from each of the remaining sequences to form all possible triplets. For a triplet, the hamming distance between any two substrings is not more than 2*d*. From the triplets, candidate motifs are generated and inserted into hash tables associated with the corresponding sequence information. Finally, if a candidate motif has been associated to all the sequences in the data set, it can be output as a true motif. Due to the generation of candidate motifs and the hashing process, iTriplet requires a large amount of memory to store information when dealing with longer and weaker motifs.

Other motif discovery algorithms have also been proposed, see for example: [10-26] etc. In this paper, we introduce a novel algorithm, RecMotif, using the concepts of reference sequence and reference vertex/substring/word. That is, if there are *m* sequences in the data set, we use the first *x* sequences as the reference sequences for the remaining *m-x* sequences, with *x* increased from 1 to *m*. For each substring in sequence *x*+1, to be a reference vertex for the next *m-x-*1 sequences, it must satisfy some measure, such as hamming distances with all current reference vertices from the first *x* sequences. If the reference vertex selection operation can reach the last sequence, a clique of motif instances of size *m* has been found. From the clique, the true motif can be obtained by alignments.

## Earlier work

### Notations

Let the DNA alphabet Ω*=*{*A, C, G, T*}*,* the length of the motif to be discovered is *l* and the number of mutations allowed is *d.* Let *S=*{*S_i_|i*=0, 1, *...*, *m-*1} be a set of *m* DNA sequences with *S_i_* having length *n_i_>l.* Sequence *S_i_*=(*s_i_,*_0_, *s_i_,*_1_, *...*, *s_i,n_i__*_-1_), where *s_i,j_*∈â„¦.

A sliding window of length *l* is used to obtain all the possible *l*-mer vertices (or substrings) of sequence *S_i_*. Put all the vertices from *S_i_* in the set *P*_0_*_,i_=*{*v_i,j_|j=*0, 1, *...*, *n_i_-l*}. Let *|P*_0_*_,i_|* denote the number of vertices in *P*_0_*_,i_*.

Each vertex is identified according to its starting position *j* in sequence *S_i_*, i.e. *v_i,j_*=(*s_i,j_*, *s_i,j_*_+1_, *...*, *s_i,j+l_*_-1_).

Let *D*(*v*_*i*_1___,__*j*_1__*,v*_*i*_2___,__*j*_2__) denote the hamming distance of two length *l* vertices *v*_*i*_1___,__*j*_1__ and *v*_*i*_2___,__*j*_2__. We have:(1)

where for α, β∈Ω,(2)

Moreover, the type of the data sets we consider is OOPS. That is, in such data sets, there will be exactly One Occurrence of the motif instance Per Sequence for the true motif.

### Background and inspiration

The concepts of reference sequence and reference word/vertex/substring have been used in DPCFG, PMSprune, iTriplet and MULTIPROFILER [17] etc.

These algorithms usually select one or two sequences as the reference(s). DPCFG uses the vertices selected according to the reference vertices to construct lists of motif instances. PMSprune uses the substrings in the reference sequence to generate new strings (candidate motif) in its neighborhood. iTriplet uses the reference substrings to generate candidate motifs with another selected substring. MULTIPROFILER uses the reference word to select substrings from the other sequences and try to recover the structure of the true motif using them.

Specifically in DPCFG, a reference sequence is selected at random and all the non-reference vertices from *S* are divided into different sets according to each of the reference vertices. Suppose *S*_0_ is selected as the reference sequence. The sets are obtained as follows.

Firstly, for each reference vertex *v*_0,*r*_0__ in *S*_0_ (i.e. *P*_0,0_), define *P*_1,_*_k_* as the set of the selected vertices from *P*_0,_*_k_*, where *k*=1, 2, ..., *m*-1, *r*_0_∈{0, 1, ..., *n*_0_-*l*}.

Initially, for each *v*_0*,r*_0__, *P*_1_*_,k_* is set as null. Then for each *v_k,j_k__* from *P*_0*,k*_, put it in *P*_1*,k*_ if *D*(*v*_0,*r*_0__,*v*_*k,j*_*k*__)*≤*2*d*, where *_j_k__*=0, 1, ..., *n_k_-l.* (Note that if the maximum distance allowed between a motif instance and the true motif is *d*, the maximum distance between any two motif instances can be 2*d*. Therefore, if the reference vertex is a motif instance, all the other motif instances will appear in the corresponding sets: *P*_1*,k*_ for 1*≤k≤m*-1.)

With these sets at hand, DPCFG initializes a list of size 1 with the current reference vertex in *P*_0,0_. Then it constructs lists for each of the vertices in *P*_1,*k*_ using lists constructed for the vertices in *P*_1,*k*-1_, *k*=1, 2, ..., *m*-1. In detail, a list *L_v_k,j_k___* for a vertex *v_k,j_k__* from *P*_1*,k*_ will be created using *L*_*v*_*k*-1, *j*_*k*-1___ if *D*( *v_k,j_k__*,*v*_*k*-1*,j*_*k*-1__)*≤*2*d*. A further checking process will be applied to determine if there is a need to maintain *L_v_k,j_k___*. Finally, any list of size *m* consists of a clique of motif instances of the same size. In the clique, all pair-wise elements have hamming distance not more than 2*d*.

It can be seen that a reference sequence or a few reference sequences together with a motif construction strategy has shown success. Based on this observation, we proposed the algorithm RecMotif, which is introduced in following section.

## Method

### RecMotif

If the motif instance in *P*_0,0_ is selected as the reference vertex, all the other motif instances must appear in the corresponding *P*_1,*k*_, *k*=1, 2, ..., *m*-1. As we do not know which vertex is the motif instance in advance, we will have to test all of the vertices in *P*_0,0_.

RecMotif further extends the idea of reference sequence and reference vertex. That is, it uses each of the vertices in *P*_1,1_ as the reference vertex to select vertices from *P*_1*,k*_ into correspondingly defined sets: *P*_2*,k*_, *k*=2, 3, *...*, *m*-1. Again, if the selected reference vertex in *P*_1,1_ is a motif instance, all the remaining motif instances must appear in *P*_2,*k*_. Then it uses vertices in *P*_2,2_ as the reference vertices to select vertices from *P*_2,*k*_, *k*=3, 4, *...*, *m*-1. The algorithm continues in this way until the second last sequence.

During the selection process, all currently selected reference vertices will form a search path. The path continuation condition is: if and only if all the remaining sequences have at least one vertex that is within *2d* with all the current reference vertices. Otherwise, the algorithm substitutes the last reference vertex with another one from the same set with it to form a new path. If there are no more new vertices in the corresponding set, the algorithm deletes the last reference vertex and backtracks to the last second reference vertex and finds a substitute for it. The deletion, backtracking and substitution process is repeated until the path continuation condition is satisfied.

Now we explain the details about RecMotif. RecMotif takes all the *l*-mer substrings from *S* as input. In a general way, we define *P*_*i*+1,*k*_ as the set of the selected vertices from *P_i,k_* according to reference vertices in *P_i,i_*, *i*=0, 1, *...*, *m*-2, *k*=*i*+1, *i*+2, *...*, *m*-1. Note that as RecMotif deals with each reference vertex sequentially, the set *P_i,k_* can be used repeatedly, which reduces the memory requirement.

For each *v_i,r_i__* in *P_i,i_*, *P_i_*_+1_*_,k_* is set as null. Then for each *v_k,j_k__* from *P_i,k_*, put it in *P_i_*_+1_*_,k_* if *D* (*v_i,r_i__, v_k,j_k__*)*≤*2*d*. The pseudo-code for the RecMotif algorithm is shown in Algorithm 1. The RecMotif algorithm works in a recursive way to complete the selection process. In the process, the selected reference vertex/veritces will be inserted into the set: *INSTANCE.* If *i=m,* the path formed by elements in *INSTANCE* indicates a *m*-clique. From the clique, the consensus motif can be obtained by alignment.

With the initial sets: *P*_0*,k*_, *k*=0, 1, *...*, *m-*1, RecMotif starts from *i*=0. That is, RecMotif(0), i.e. the first reference sequence is *S*_0_. When RecMotif(*m*) is processed, it indicates that a clique of size *m* have been found.

**Figure 1:**
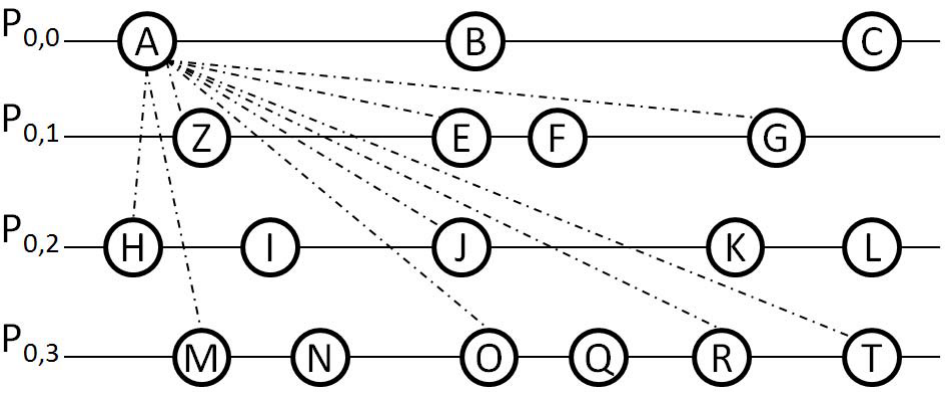
**RecMotif example**. This figure gives an example for explaining the process of RecMotif.

An example about how RecMotif works is shown in Figure 1 with *m*=4. Assume *P*_0,0_={*A*, *B, C*}, *P*_0,1_*=*{*Z, E, F, G*}*, P*_0,2_*=*{*H, I, J*, *K, L*}, *P*_0,3_*=*{*M, N, O, Q, R, T*}. Each of the capital letters stands for a vertex (substring) of length *l*.

Moreover, assume the following relationships on pair-wise hamming distances of vertices involved in the explanation of the example. Let *A*_2*d*_*=*{*Z, E, G, H, J, M, O, R, T*} indicates that vertex *A* has hamming distances not more than 2*d* with vertices *Z, E, G, H, J*, *M, O, R, T* and more than 2*d* with the other vertices not mentioned. Similarly, we assume *Z*_2*d*_*=*{*A, H, J*, *M*, *T*}; *E*_2*d*_*=*{*A*}*; G*_2*d*_*=*{*A, T*}*; H*_2*d*_*=*{*A, Z*}*; J*_2*d*_*=*{*A, Z, M, T*}*; M*_2*d*_*=*{*A, Z, J*}*; O*_2*d*_*=*{*A*}*; R*_2*d*_*=*{*A, H*}*; T_2*d*_=*{*A, Z, G, J*}*.*

### Algorithm 1 RecMotif (*i*), *i*≥0

1: **bool***is Valid*;

2: **if***i<m***then**

3: **for** each *v_i,r_i__*∈*P_i,i_***do**

4: *is Valid* = *TRUE*;

5: **for** each *P_i_*_+1_,*_k_*, *i*+1*≤k≤m-*1 **do**

6: *P*_*i*+1,*k*_←*NULL*;

7: **for** each *v_k,j_k__*∈*P_i,k_***do**

8: **if** D (*v_i,r_i__,v_k,j_k__*)≤2*d***then**

9: *P*_*i*+1_,*k*←*v_k,j_k__*;

10: **end if**

11: **end for**

12: **if** |*P_i_*_+1,_*_k_*|=0 **then**

13: *is Valid*=*FALSE*;

14: **break**;

15: **end if**

16: **end for**

17: **if***is Valid***then**

18: *INSTANCE[i]*=*v_i,r_i__*;

19: RecMotif (*i*+1);

20: **end if**

21: **end for**

22: **else**

23: **output** the clique: *INSTANCE[j], j=*0, 1, *...*, *m-*1;

24: **end if**

With the above assumptions, we begin to proceed in RecMotif(*i*) with *i*=0. Firstly, vertex *A* from *P*_0,0_ is selected as the reference vertex. *P*_1,1_ is set as null.

**Figure 2:**
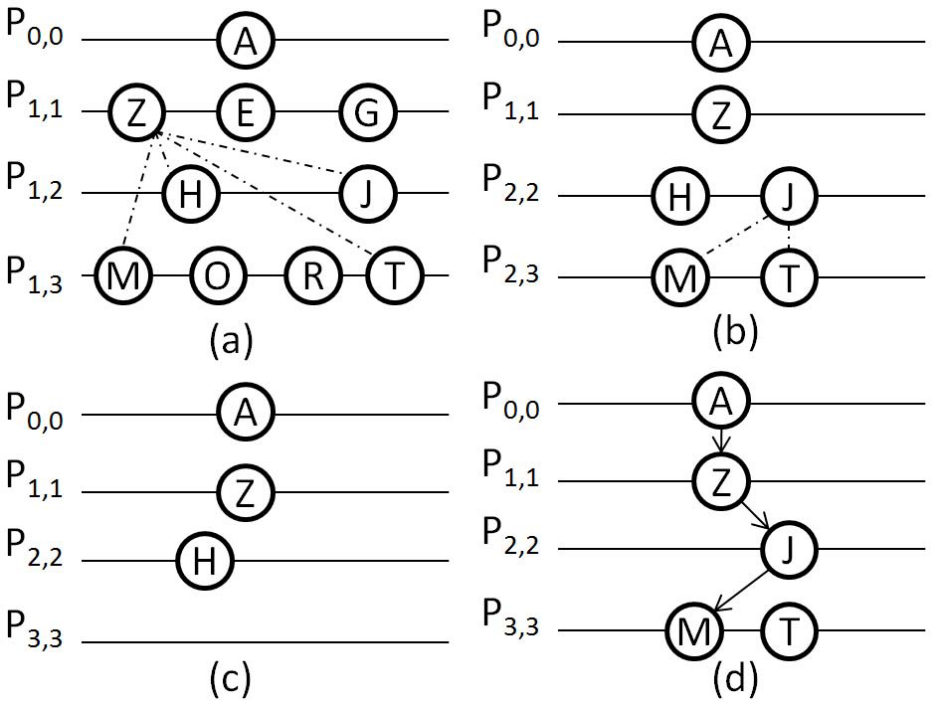
**Example for processing vertex A**.This figure gives an example for processing a vertex A in the example of Figure 1.

All vertices in *P*_0,1_ as shown in Figure 1 will be checked if they are within 2*d* with *A.* According to the assumptions, vertex *A* is within 2*d* with *Z, E, G* from *P*_0,1_ (connected by dotted lines in Figure 1). Thus we can obtain *P*_1,1_*=*{*Z, E, G*}*.* Similarly, we can obtain *P*_1,2_*=*{*H, J*} and *P*_1,3_={*M*, *O, R, T*} as has been shown in Figure 2(a).

As can be seen, for vertex *A,* we can find vertices that have hamming distance not more than 2*d* with it from all other sets: *P*_0,1_, *P*_0,2_ and *P*_0,3_, therefore it could be a motif instance. We set *INSTANCE[0]=A* and begin to proceed in RecMotif(*i*) with *i=*1*.* (RecMotif(0) is suspended temporarily. Moreover, note that for each *P_i_*_+1_*_,k_*, *i*=0, 1, *...*, *m-*1, *k*=*i*+1, *i* +2, *...*, *m-*1, it must be set as null each time before selecting vertices from the corresponding *P_i,k_*. In the following explanation, we will not repeat the descriptions on such operations.)

Next, vertex *Z* from *P*_1,1_ is selected as the reference vertex. All vertices in *P*_1,*k*_, *k*=2, 3, will be checked.

Vertex *Z* is within 2*d* with *H, J* from *P*_1,2_ and *M*, *T* from *P*_1,3_, as shown by the links in Figure 2(a). That is, we can obtain *P*_2,2_*=*{*H, J*} and *P*_2,3_*=*{*M, T*}*,* as shown in Figure 2(b). Also, it is possible for vertex *Z* to be a motif instance. We set *INSTANCE[1]=Z* and begin to proceed in RecMotif(*i*) with *i* being 2 (RecMotif(1) is suspended temporarily).

Next, vertex *H* from *P*_2,2_ is selected as the reference vertex. All vertices in *P*_2,_*_k_*, *k*=3, will be checked. As *H* has no vertex from *P*_2,3_ that has hamming distance not more than 2*d* with it, current *P*_3,3_ is null. Thus *INSTANCE[2]* will not be set as *H.* In other words, there will be no path to form a *m*-clique involving vertex *H* with the current elements in *INSTANCE,* as shown in Figure 2(c). We have to continue the process in RecMotif(2) with another vertex from *P*_2,2_.

Next, vertex *J*, which is the next vertex to be checked in the same set *P*_2,2_ as *H,* is selected as the reference vertex. All vertices in *P*_2,_*_k_*, *k*=3, will be checked. As *D*(*J, M*)*≤*2*d* and *D*(*J,T*)*≤*2*d, P*_3,3_ is obtained as {*M*, *T*}*.* As it is possible for vertex *J* to be a motif instance, we set *INSTANCE[2]=J* and begin to proceed in RecMotif(*i*) with *i*=3 (RecMotif(2) is suspended temporarily).

Next, vertex *M* from *P*_3,3_ is selected as the reference vertex. As *i*=*m-*1, indicating vertex *M* is a vertex in the last sequence thus there are no more vertices to be checked for vertex *M.* We directly set *INSTANCE[3]=M* and begin to proceed in RecMotif(*i*) with *i*=4 (RecMotif(3) is suspended temporarily). Now, *i* is equal to *m*. This means that a path to form a *m*-clique is formed, as shown in Figure 2(d). The elements to form the clique have been stored in *INSTANCE* as: *A, Z, J*, *M.*

At this point, the process for vertex *M* has been completed (i.e. current RecMotif(4) completed). We return to proceed in RecMotif(3). The process that was carried out for vertex *M* is repeated for vertex *T* from *P*_3,3_. Using this process we can obtain another clique as: *A, Z, J, T* from *INSTANCE* according to the assumptions.

As vertex *T* is the last vertex in *P*_3,3_, the RecMotif(3) process is completed. Thus we return to RecMotif(2). As vertex *J* is the last vertex in *P*_2,2_, the RecMotif(2) process is completed. Thus we return to the RecMotif(1) process. Hitherto, the whole process for vertex *Z* from *P*_1,1_ has been completed.

As there are still vertices *E, G* in *P*_1,1_, we repeat similar processes (to that for *Z*) for *E* and *G* respectively. When the process for vertex *G* is completed, it means that the process of RecMotif(1) has been completed. Thus we finally return to the process of RecMotif(0).

At this point, the process for vertex *A* has been completed. As there are still vertices *B, C* in *P*_0,0_, we repeat similar processes (to that for *A*) for *B* and *C* respectively. When the process for vertex *C* is completed, it means that the RecMotif(0) process has been completed. That is, the whole algorithm is completed with all possible cliques found (if any).

Next, we will give the analysis on time and space complexities, as discussed in the following section.

### Running time and space analysis

#### Time complexity

Assume the sequence length is *n* and there are *m i.i.d* sequences in total. Define *p* as the probability that the hamming distance between two random vertices of length *l* from the data is not more than 2*d. p* is calculated by Equation 3. Note that *p* reflects the weakness of the motifs to be discovered [4,8].(3)

We now analyze the time complexity of RecMotif by estimating the number of hamming distance calculations.

For a data set of *m* sequences, |*P*_0,*k*_0__|=*n-l*+1, where 0*≤k*_0_*≤m-*1; correspondingly, for the sets to store selected vertices, |*P _i,k_i__*|=(*n-l+*1)*p* with 1*≤k*_1_*≤m-*1, *...*, |*P_m-_*_1,__*k*_*m*-1__|=(*n-l*+1)*p*^*m-*1^ with *m-*1*≤k_m-_*_1_*≤m-*1.

Suppose *x_i_,* 0*≤i≤m-*2, is the number of hamming distance calculations that are expected for using all vertices in each *P_i,i_* to select vertices in *P_i,k_*, *i*+1*≤k≤m-*1.

Specifically, for each vertex in *P*_0,0_, we have to check all vertices in *P*_0,*k*0_, 1*≤k*_0_*≤m-*1. Thus there can be (*m -* 1)(*n - l +* 1) calculations. As there are *n*-*l*+1 such vertices appearing in *P*_0,0_, *x*_0_*=*(*m -* 1)(*n - l* + 1)^2^.

For each vertex in *P*_1,1_, we have to check all vertices in *P _i,k_i__*, 2*≤k*_1_*≤m-*1. Thus there can be (*m -* 2)(*n - l +* 1)*p* calculations. As there are (*n - l +* 1)(*n - l + 1*)*p* such vertices appearing in *P*_1,1_, *x*_1_=(*m-*2)(*n-l*+1)^3^*p*^2^.

Generally, for each vertex in *P_i,i_*, *i*=0, 1, *...*, *m-*2, we have to check all vertices in *P_i,k_i__*, *i*+1*≤k_i_≤m-*1. Thus there can be (*m - i -* 1)(*n - l* + 1)*p^i^* calculations. As there are  such vertices appearing in 

Thus the total number of calculations can be:. Approximately the time complexities of RecMotif with different relationships between *p* and *n* are shown in Table 1 (for more details, please refer to the additional materials). The time and space complexities of RecMotif and several related algorithms are shown in Table 2 (the analysis of RecMotif’s space complexity is given in the next section).

**Table 1:** Time complexities of RecMotif.

Range of *p*	*O*(*•*)	Range of *p*	*O*(*•*)
(0,0.035]	*mn*^2^	(0.32,0.38]	*mn*^5^*p*^6^
(0.035,0.10]	*mn*^3^*p*^2^	(0.38,0.43]	*mn*^5^*p*^3^
(0.10,0.18]	*mn*^3^*p*	(0.43,0.47]	*mn*^6^*p*^8^
(0.18,0.26]	*mn*^4^*p*^4^	(0.47,0.51]	*mn*^6^*p*^4^
(0.26,0.32]	*mn*^4^*p*^2^	(0.51,0.54]	*mn*^7^*p*^10^

This table shows the complexities of RecMotif with different *p* and *n*.

**Table 2:** Algorithm complexities.

Algorithm	Complexity	
Time	Space

PMSprune	*O*(*mn*^2^*N*(*l, d*))	*O*(*mn*^2^)
iTriplet	*O*(*mn*^3^*pl*^3^*d*^2^)	*O*(*N*(*l, d*))
DPCFG	*O*(*mn*^3^*p*^2^ + *n*^*m*^*p*^3*m-*6^)	*O*(*mn*^4^*p*^3^)
RecMotif	See Table 1	*O*(*m*^2^*n*)

This table shows time and space complexities of PMSprune, iTriplet, DPCFG and RecMotif.

*note,

When *p<*0.12 (with *n*=800), the dominating factor of DPCFG is *mn*^3^*p*^2^*.* This is approximately the same as RecMotif. However, as the time of DPCFG is contributed by list maintenance and hamming distance calculation, there is more overhead involved (by list maintenance) in practice. When *p>*0.12, the dominating factor of DPCFG is *n^m^p*^3*m*-6^ which is larger than RecMotif. For PMSprune, its running time grows with *n*^2^ for small *N*(*l, d*)*,* which is better than RecMotif. However, for large *l* and *d* which involves large *N*(*l, d*)*,* the running time of PMSprune grows exponentially (even when the corresponding *p* is small). iTriplet is also affected by *l* and *d* with the same reason as PMSprune.

#### Space complexity

As has been discussed, we have |*P*_0,*k*_0__|=*n - l* + 1 vertices for each *k*_0_ with 0*≤k*_0_*≤m -* 1, |*P*_1,_*k*_1_|=(*n - l +* 1)*p* vertices for each *k*_1_ with 1*≤k*_1_*≤m -* 1, *...*, (*n - l + 1*)*p^m-^*^1^ vertices for *P_m_*_-1_*_,m_*_-1_ and *m* vertices for *INSTANCE.*

Generally, for *P_i,i_*, *0≤i≤m -* 1, there are (*m - i*)(*n - l +* 1) *p^i^* vertices to be stored. Thus the total space needed is:  As *p^i^* is less than 1, thus the total space should be less than . Thus the approximate space complexity is *O*(*nm*^2^)*.*

From Table 2 it can be seen that RecMotif consumes much smaller space than other algorithms. The space complexity of iTriplet increases exponentially as *l* or *d* increases. DPCFG consumes much larger space than RecMotif. This will result in DPCFG having difficulties in dealing with motifs such as (24, 8). While PMSprune requires less memory than DPCFG, it is still larger than RecMotif (as *n*»*m*)*.*

Next, we will carry out experiments to compare the performances of the above algorithms in practice.

## Results and discussions

As the parameters *n* and *p* will influence the weakness of a motif, we will therefore first discuss experiments with synthetic data generated with different values of *n* and *p.* Then we will apply RecMotif to real biological data set to show its feasibility in practice. All the following experiments are carried out on a PC with a 2.66 GHz processor and 3 GB RAM.

### Synthetic data

For synthetic data, the sample sequences are generated as independent and identically distributed (i.i.d) [1]. And the positions (0, 1, *...*, *n-l*) where the motif instances are planted in the sequences are also selected at random. Recall Rate *R_r_* are used as the indicator, defined in Equation 4.(4)

where *K_n_* (|*K_n_*|=*ml*) is the set of the known base positions and *P_d_* is the set of the predicted base positions for the motif. *R_r_* indicates how many of the planted positions have been discovered. If *R_r_*=1, it means that all the planted positions have been discovered.

#### Comparisons on increased sequence length

In this section, we fixed the motif (*l*, *d*) as (15, 4) and increased the sequence length *n* from 600 to 2000.

**Figure 3:**
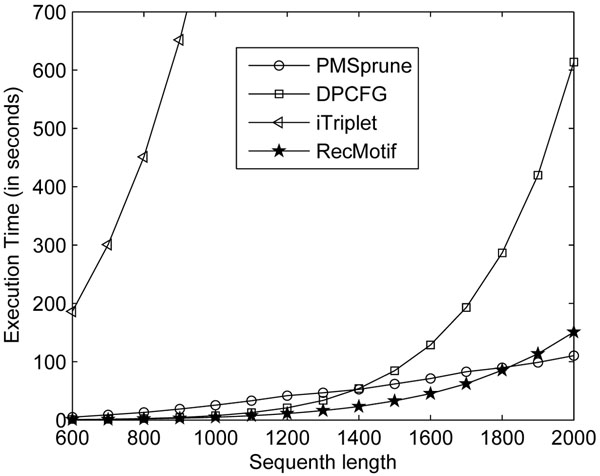
**Effects of sequence length n on execution time on model (15, 4)**. This figure shows for MCP, how the execution times of PMSprune, iTriplet, DPCFG and RecMotif change with increasing n.

For each setting, 10 i.i.d. data sets are generated, each containing 20 sequences. All the algorithms discovered the motif with *R_r_*=1. Thus we compare their performances in terms of execution time, as shown in Figure 3. Note: as iTriplet consumes much more time than the other algorithms when *n>*900, only a part of execution times is shown.

Figure 3 shows that when *n<*1800, RecMotif can achieve the best execution time over all the other algorithms. RecMotif consumes less than half the execution time of DPCFG for data sets with sequences longer than 1300. For longer sequences such as 2000, RecMotif consumes only one-fourth the execution time of DPCFG.

One of the reasons why DPCFG runs much slower than RecMotif is that the lists construction is dynamic which involved many memory operations. Comparatively, RecMotif just proceeds to find a path and requires the recording of not more than *m* elements only. With less overhead in maintaining intermediate information, RecMotif is able to achieve better time performance.

For fixed (*l*, *d*), as *n* increases, PMSprune exhibits linear increase in execution time. When sequence length is longer than 1800, PMSprune shows the best execution time. But the execution time of RecMotif is still comparable to PMSprune. The difference is less than one minute.

PMSprune performs better than RecMotif and the other algorithms under this setting because the value of *l* is relatively short, that is, the neighborhood *N*(*l*, *d*) of the sample substrings are very small. In other words, the small search space for the current settings makes PMSprune faster. As *l* increases, however, the execution time of PMSprune will grow exponentially, as will be shown in the next section.

#### Comparisons on fixed probability p

In this section, we carried out comparisons on the probability *p* calculated using Equation 3, as it can reflect the weakness of the true motif.

We fixed the value of *p* around 0.05, which is approximately the same as the (15, 4) settings. The experimental results are shown in Table 3. All execution times are averaged from 10 i.i.d. data sets with *n*=600, *m*=20.

**Table 3:** Effects of (*l, d*) on execution time.

(*l*, *d*): *p*	Algorithm			

	DPC-FG	PMS-Prune	iTr-iplet	Rec-Motif
(12, 3): 0.054	0.825	1.63	173.6	0.630
(15, 4): 0.057	0.673	5.22	189.2	0.703
(18, 5): 0.057	0.596	16.9	230.4	0.700
(21, 6): 0.056	0.532	46.5	250.0	0.677
(24, 7): 0.055	0.475	80.2	291.5	0.585
(27, 8): 0.053	0.432	137.1	354.4	0.633
(30, 9): 0.051	0.394	242.9	443.6	0.629
(33, 10): 0.048	0.365	405.2	553.8	0.556
(36, 11): 0.046	0.329	651.8	1419	0.484
(39, 12): 0.044	0.311	1056	2779	0.500
(42, 13): 0.042	0.286	1842	2895	0.483
(44, 14): 0.063	0.674	-o	-e	0.971
(47, 15): 0.059	0.577	-o	-e	0.921
(50, 16): 0.055	0.520	-o	-e	0.832

This table shows how the execution times of algorithms, including PMSprune, iTriplet, DPCFG and RecMotif are influenced by the values of *l* and *d* with approximately fixed p.

*note, -o: over 5 hours; -e: error on memory allocation. Time unit: seconds

From Table 3, it can be seen that the performances of PMSprune and iTriplet are very sensitive to the motif length *l*. When *l* increases, the execution times of PMSprune and iTriplet increase exponentially. In addition, iTriplet may encounter memory allocation error when handling longer motifs such as (44, 14).

This happens because when *l* becomes longer, the neighborhood *N*(*l*, *d*) of the sample substrings grows exponentially. That is: for PMSprune, it needs to check more strings; for iTriplet, it has to maintain many candidate motifs in the hash table.

Meanwhile, it can be seen that RecMotif and DPCFG exhibit stable and comparable execution times, which are little affected by the motif length and significantly better than those of PMSprune and iTriplet.

#### Comparisons on different probability p

**Figure 4:**
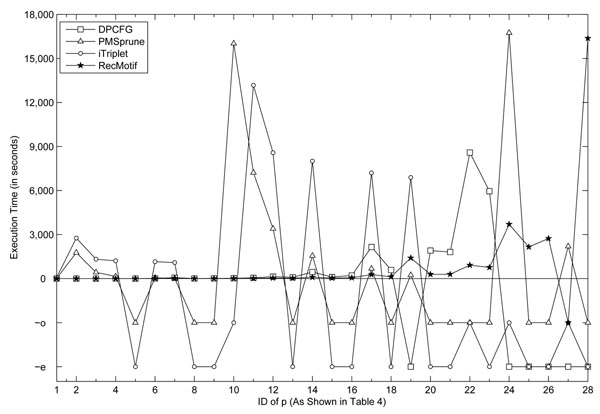
**Effects of increasing p (with related (l, d)) on execution time** This figure shows how the execution times of algorithms, including PMSprune, iTriplet, DPCFG and RecMotif, change with the parameter *p*.

In this section, we set the value of *p* from 0.029 to 0.285 by varying the values of *l* and *d* as shown in Table 4 and carry out experiments on different algorithms with them. The results are shown in Figure 4. All the execution times are averaged from 10 i.i.d. data sets with *n*=600, *m*=20.

**Table 4:** Different motifs for increasing *p*.

ID	*p*	(*l, d*)	ID	*p*	(*l, d*)
1	0.029	(28, 8)	15	0.163	(50, 17)
2	0.086	(29, 9)	16	0.167	(36, 12)
3	0.096	(23, 7)	17	0.175	(19, 6)
4	0.101	(20, 6)	18	0.181	(33, 11)
5	0.103	(40, 13)	19	0.190	(16, 5)
6	0.107	(17, 5)	20	0.197	(30, 10)
7	0.112	(14, 4)	21	0.206	(41, 14)
8	0.112	(37, 12)	22	0.214	(27, 9)
9	0.119	(34, 11)	23	0.223	(38, 13)
10	0.128	(31, 10)	24	0.234	(24, 8)
11	0.139	(28, 9)	25	0.242	(35, 12)
12	0.149	(25, 8)	26	0.260	(54, 19)
13	0.155	(39, 13)	27	0.283	(18, 6)
14	0.162	(22, 7)	28	0.285	(40, 14)

This table shows the values of *l* and *d* with increasing *p*.

From Figure 4, it can be seen that as *p* increases, the execution times of RecMotif and DPCFG become longer with relatively smooth trends. At the same time, the execution times of PMSprune and iTriplet also become longer. The execution times of iTriplet are much longer than RecMotif.

PMSprune is sensitive to the motif length *l* as well as the probability *p*. If the difference in the values of *p* of the two models is small, the performance is determined by the motif length. However, when the difference in the values of *p* is large, *p* will determine the performance.

For example, when *p*=0.112 for models (14, 4) and (37, 12), PMSprune consumes more than 5 hours on the latter model compared to 20 seconds on the former. This is the same as the comparisons in the last section.

For models (24, 8) and (25, 8) where their values of *p* show a relatively larger difference, PMSprune spent much more time on the weaker one (24, 8) although the motif length of the latter is longer.

Moreover, PMSprune requires over 5 hours when handling motifs with *l≥*30 and *p>*0.1. This reflects that it is unable to handle longer motifs of more degenerate models.

Different from PMSprune, the longer the motif the less time is required by RecMotif when the values of *p* for the two models are large but the relative difference of the two *p* values is small. Taking models (22, 7) and (50, 17) as an example, RecMotif shows a lower computational time on the latter model (90 seconds for the former and 40 seconds for the latter).

The difference happens because PMSprune has to check all the strings in the neighborhood *N*(*l*, *d*) of a reference substring from a reference sequence. When *l* increases, more strings have to be generated and checked, which incurs more execution time.

As RecMotif is a sample-driven algorithm, it only has to manipulate substrings in the data set, i.e. (*n - l* + 1)*m* substrings. For a data set with fixed *n* and *m*: when *l* is short, two length *l* substrings have a higher chance to be 2*d*-neighbors; when *l* is long, two length *l* substrings have a lower chance to be 2*d*-neighbors.

This is because the number of different strings of length *l* is determined by 4*^l^* and thus the longer the string the lower is the chance for it to be repeated exactly in a data set with a finite number of substrings. Likewise, the 2*d*-neighbors of a string also have a lower chance to be included in the data set when *l* is larger.

Thus less random cliques of small sizes will be formed. This is why RecMotif requires a lower execution time on such models.

Figure 4 also shows that when *p* is below 0.167 (corresponding to ID 16), DPCFG requires comparable execution time with RecMotif. However, when *p>*0.167, DPCFG runs much slower than RecMotif (more than 4 times the execution time of RecMotif). In addition, when *p>*0.23, DPCFG runs out of memory because of the need to maintain many random cliques in the intermediate steps.

RecMotif is capable of handling weaker and longer motifs than other algorithms, such as (24, 8) and (40, 14). For model (24, 8), RecMotif requires only one-fourth the execution time of PMSprune (while DPCFG encounters memory allocation errors and iTriplet requires more than 5 hours to produce the results). For model (40, 14), RecMotif can produce the results within 5 hours while all the other algorithms failed because of memory error or failing to produce a result within the execution time limit of 5 hours.

However, for model (18, 6) with *p* as 0.283 which is less than that of the model (40, 14), only PMSprune can produce the results within reasonable time while RecMotif and the other algorithms failed. iTriplet can produce results on this model with an execution time of more than 10 hours. DPCFG has memory allocation errors while RecMotif takes several days to produce the results. RecMotif failed due to the reason that there are too many random substrings for shorter and weaker models to check in the sample data as analyzed previously. Therefore, the capability of RecMotif to deal with shorter and weaker motifs (such as (13, 4), (15, 5) etc) still needs to be improved.

The general view of Figure 4 shows that the two pattern-driven algorithms PMSprune and iTriplet show similar performances trends as *p* increases. Besides *p*, their performances are also sensitive to motif lengths. Meanwhile, RecMotif and DPCFG, driven by sample substrings, also show similar performance trends as *p* increases except that DPCFG has memory allocation errors for large values of *p*. Overall, RecMotif is more scalable on both the value *p* and the motif length *l* compared to the other algorithms.

### Biological data

To verify if RecMotif can be used to discover motifs in biological data, we tested it on several TFBS data sets. These data sets are *E.coli CRP*[10], *Pre-proinsolin*, *DHFR* and *c-fos*[17], and *LexA*[14].

To carry out experiments, we assume these data sets are with the OOPS condition. In practice, as we do not have any information about the model (*l*, *d*) of the true motif in the data, we change the value of *l* starting from 6 for RecMotif. For each *l*, we tested different values of *d* (*d < l/*2) to check if any cliques can be discovered. By aligning the cliques, we check if the target motif (i.e. the published motif in that data set) had been discovered. The motifs discovered by RecMotif are shown in Table 5.

**Table 5:** Results of RecMotif on biological data

Data	Discovered	Model	Published
*c-fos*	***ccatattaggacatct***	(16, 3)	ccatattaggacatct
*DHFR*	***ttcgcgccaaact***	(13, 2)	ttcgcgccaaact
*E.coli CRP.*	***tgtgaaxxagxtcaca*tt**	(18, 6)	tgtgaxxxxgxtcaca
*LexA*	***tactgtatataxatacagta***	(20, 5)	tactgtatatatatacagta
*Pre-proinsulin*	***cctcagcccc***	(10, 2)	cctcagcccc,
	***agacccagca***		agacccagca,
	***ccctaatgggcca***	(13, 2)	ccctaatgggcca

This table shows the results of RecMotif on real biological data.

*note: ’x’ in the consensus means all bases appear with opportunity less than 50 percent

Table 5 shows that RecMotif is applicable in real DNA sequences. Among the data, *E.coli CRP* includes 18 sequences of length 105 with each containing at least one motif instance. The motif it contains is weaker (that five positions on it cannot obtain dominant bases). By setting the (*l*, *d*) as (18, 6), RecMotif discovered the published motif in less than 1 second. While with the same setting to find the motif, iTriplet requires about 4 minutes, DPCFG requires about 30 seconds and PMSprune requires about 10 seconds. RecMotif has shown relatively better performance compared to these algorithms.

## Conclusions

DNA motif discovery is a fundamental problem in computational biology. Although many deterministic algorithms have been proposed for it, these algorithms usually become time-prohibitive when searching for longer and weaker motifs or consume substantial memory resources (even running out of memory). In this paper, we proposed an algorithm, RecMotif, which exhibits time and memory efficiency and guarantees that all motifs can be discovered for specific (*l*, *d*) settings. RecMotif uses the tenet of constructing cliques recursively using the sample substrings in the data based on the concept of reference sequence/vertex. The process of RecMotif is that it uses the selected reference vertices from the first *x* reference sequences to select new reference vertices in the remaining sequences. With *x* gradually increased, if new reference vertices can be selected from all the remaining sequences, the selection is continued. Finally if *x* equals *m*, it means a clique of motif instances has been discovered.

RecMotif requires only a space complexity of *O*(*nm*^2^) for processing. As *m* and *n* are fixed for each run on a certain data set, the whole process of RecMotif consumes almost constant memory. Not only can this reduce execution time on memory operations but it also can prevent memory allocation errors for weaker motifs.

The performance of RecMotif is affected by the background sequence *n* and the probability *p*. For a fixed *p*, the longer the sequences the more execution time RecMotif requires. This is the same with all the other algorithms compared. Meanwhile, if *l* and *d* are changed but *p* is preserved, it will not lead to longer execution time for RecMotif. This distinguishes it from pattern-driven algorithms such as PMSprune. As *p* increases, the execution time of RecMotif grows slower than that of DPCFG and it exhibits better performance in handling longer and weaker motifs than DPCFG, iTriplet and PMSprune.

Increasing the values of *n* and *p* will lead to a longer execution time for RecMotif. This is because when *n* and *p* become larger, more random vertices in each sequence are selected as candidate reference vertices. This results in many more combinations of random selection paths, i.e. intermediate cliques of small sizes. Therefore, to eliminate these random cliques, more comparisons (incurring execution time) are needed to make between the real motif instances and the random vertices.

Overall, RecMotif has been shown to exhibit better performance compared to the other algorithms tested. However, for shorter and weaker (*l*, *d*) motifs such as (18, 6) with *n*=600 and *m*=20 that can be solved by PMSprune, RecMotif has difficulty in producing results within reasonable execution time. Thus future work is still needed to improve RecMotif on solving such problems.

## Availability

Source code of RecMotif can be requested by emails.

## Competing interests

The authors declare that they have no competing interests.

## Authors contributions

HQS, MYHL, WJH and JCR conceived, wrote and revised the manuscript. HQS designed and developed the algorithm and carried out the experiments.
